# Pathogenicity, immunogenicity, and multi-organ tissue tropism of a novel variant infectious bursal disease virus in specific pathogen-free chickens

**DOI:** 10.14202/vetworld.2026.591-603

**Published:** 2026-02-17

**Authors:** Mohd Hair-Bejo, Mazlina Mazlan, Tein Hock Be, Chong Hao Han, Norfitriah Mohamed Sohaimi, Chidozie Clifford Ugwu

**Affiliations:** 1 Faculty of Veterinary Medicine, Universiti Putra Malaysia, 43400 Serdang, Selangor, Malaysia; 2 Institute of Bioscience, Universiti Putra Malaysia, 43400 Serdang, Selangor, Malaysia; 3 Department of Animal Science and Technology, Federal University of Technology, Owerri 460114, Imo State, Nigeria

**Keywords:** infectious bursal disease, nVarIBDV, pathogenicity, specific pathogen-free chicken, bursa of Fabricius, IBD antibody, viral loads

## Abstract

**Background and Ai:**

Novel variant infectious bursal disease virus (nVarIBDV) occurs worldwide, causing significant morbidity and bursal atrophy in chickens, leading to immunosuppression and heavy economic losses. This study aimed to evaluate the pathogenicity, immunogenicity, and virus load of nVarIBDV in organs of specific-pathogen-free (SPF) chickens.

**Materials and Method:**

Sixty, 21-day-old SPF chickens were divided into Groups A and B. Birds in Group A were inoculated with 106.75 EID50/ mL of nVarIBDV (UPM1432/2019) while Group B served as a control. Four birds from Group B were sacrificed at 0 days post-inoculation (dpi). Four birds from each group were sacrificed at 1, 3, 5, 7, 10, 14, and 21 dpi. Clinical signs and serum samples were collected. Body, bursa, and spleen weights, and gross lesions were recorded. Bursa samples were collected for histological examination, while the bursa, spleen, caecal tonsils, thymus, and bone marrow were collected for the virus load determination. Data was analyzed using Student’s t-test at 95% confidence level.

**Results:**

Watery diarrhea and ruffled feathers, bursal atrophy, yellowish stain, decreased folds, firm consistency of bursa, and splenomegaly were observed in Group A. Bursal follicles were atrophied with bursal lesion scores of 4 to 5. IBD antibody titer in Group A, ranging from 6921 -13869 ELISA units at 5-21 dpi, was significantly higher (*p*<0.05) than in Group B at all timepoints. The viral load was highest in the bursa and lowest in the bone marrow and was detected from 1-21 dpi in the bursa, spleen, caecal tonsil, and thymus, and up to 7 dpi in the bone marrow with a copy number ranging from 7.111 - 12.414 log10.

**Conclusion:**

The nVarIBDV was highly pathogenic, immunogenic, and highly infective in the organs of SPF chickens. It could cause immunosuppression in chickens, exposing them to secondary infections with resultant heavy economic losses, and this report of the detection of nVarIBDV in these organs up to 21 dpi is novel.

## INTRODUCTION

The infectious bursal disease virus (IBDV) is a double-stranded RNA virus belonging to the genus Avibirnavirus within the family Birnaviridae. IBDV is a non-enveloped virion characterized by a single capsid shell with icosahedral symmetry [1]. The viral genome encodes five proteins (VP1–VP5), among which VP2 is the major structural protein and a key determinant of viral pathogenicity and antigenicity [2]. Two antigenically distinct serotypes of IBDV have been identified: serotype 1, which is pathogenic in chickens, and serotype 2, which is non-pathogenic; importantly, there is no cross-protection between these serotypes [2]. Serotype 1 viruses are further classified into three pathotypes based on virulence, namely classical IBDV (caIBDV), variant IBDV (varIBDV), and very virulent IBDV (vvIBDV) [3].

In 2017, a novel variant IBDV (nVarIBDV) with distinct genetic characteristics, clearly divergent from earlier varIBDV strains, emerged in China and was confirmed to induce marked immunosuppression in infected chickens [4]. Subsequently, antigenic variant strains showing high genetic similarity to the Chinese nVarIBDV have been reported in Japan [5], South Korea [6], and Malaysia [3]. These emerging variants are largely attributed to the high mutation rate within the hypervariable region of the VP2 protein, resulting in antigenic drift and enabling viral escape from vaccine-induced immunity [7, 8].

In general, IBDV causes an acute and highly contagious disease in young chickens, most commonly between 3 and 6 weeks of age [9]. In contrast, nVarIBDV infection is primarily associated with immunosuppression in susceptible chickens, although mortality has occasionally been reported [4]. Immunosuppressed birds are consequently more vulnerable to secondary infections, including Newcastle disease, avian influenza, and opportunistic bacterial and parasitic infections, and are also at increased risk of vaccination failure [4]. Infection with nVarIBDV is typically subclinical and is characterized by reduced body weight gain and poor feed conversion efficiency [10]. Experimental infection of specific-pathogen-free (SPF) chickens with nVarIBDV has been shown to induce whitish watery diarrhea without severe clinical signs or mortality [5, 11].

Pathological changes associated with nVarIBDV include bursal atrophy, yellowish discoloration, hemorrhage, and inflammatory exudation. Splenomegaly and an increased spleen-to-body weight ratio may be evident from 4 days post-inoculation (dpi) [4]. Thymic atrophy and muscular hemorrhage have also been reported in infected chickens [12]. Histopathological findings commonly include lymphocyte depletion, macrophage infiltration, fibrous tissue proliferation surrounding follicles, and follicular atrophy [4]. Mild to moderate degeneration and necrosis in the medullary region of bursal lymphoid follicles, accompanied by inflammatory cell infiltration, have been observed following inoculation with pathogenic field strains of nVarIBDV [13]. Additional lesions such as lymphocyte necrosis, vacuolation, cyst formation, and epithelial infolding within bursal follicles have also been documented [5].

Transmission of IBDV occurs primarily via the oral route through ingestion of contaminated feed or water, as well as via the ocular route [4]. Following entry, viral replication initially occurs in lymphoid cells of the gut-associated lymphoid tissue (GALT) [14] and the head-associated lymphoid tissues, which include the Harderian gland and conjunctiva-associated lymphoid tissue (CALT) [15]. The virus is subsequently disseminated by phagocytic cells, particularly macrophages, to secondary lymphoid organs such as the bursa of Fabricius, spleen, cecal tonsil, thymus, and bone marrow, where further replication and secondary viremia occur [16]. Viral replication within these lymphoid organs results in immunosuppression and may predispose infected chickens to fatal secondary infections [17, 18]. Although nVarIBDV exhibits a strong tropism for actively dividing precursor B lymphocytes within the bursa of Fabricius, its persistence beyond 14 dpi remains poorly defined. Moreover, while other immune organs, including the spleen, cecal tonsil, thymus, and bone marrow, may also be affected [11], information regarding viral presence and persistence in these tissues remains limited

Despite increasing reports of nVarIBDV circulation and its association with immunosuppression, significant gaps remain in understanding its pathogenic behavior under controlled experimental conditions. Most existing studies have focused on genetic characterization or short-term pathogenicity, with limited emphasis on the temporal dynamics of viral replication, tissue tropism, and persistence across multiple immune organs in SPF chickens. In particular, the duration and magnitude of nVarIBDV presence beyond 14 dpi, especially in secondary lymphoid tissues such as the spleen, cecal tonsil, thymus, and bone marrow, remain poorly defined. Furthermore, the relationship between viral load, bursal pathology, and humoral immune responses over an extended infection period has not been comprehensively evaluated. This lack of integrated data hampers a clear understanding of the mechanisms underlying nVarIBDV-induced immunosuppression, vaccine failure, and prolonged production losses, thereby limiting evidence-based refinement of IBDV control and vaccination strategies.

Therefore, this study aimed to systematically evaluate the pathogenicity, immunogenicity, and multi-organ tissue distribution of nVarIBDV in SPF chickens following experimental infection. Specifically, the study sought to (i) characterize clinical manifestations, gross and histopathological lesions, and bursal lesion severity, (ii) assess humoral immune responses through IBD antibody kinetics, and (iii) quantify viral loads in the bursa of Fabricius, spleen, cecal tonsil, thymus, and bone marrow over a 21-dpi period. By integrating pathological, immunological, and molecular findings, this study aimed to provide a comprehensive understanding of nVarIBDV infection dynamics and organ persistence, thereby generating critical evidence to inform vaccine development, challenge models, and improved control strategies against emerging variant IBDV strains.

## MATERIALS AND METHODS

### Ethical approval

All animal experiments were reviewed and approved by the Institutional Animal Care and Use Committee of Universiti Putra Malaysia (IACUC) (Approval No. UPM/IACUC/AUP-U016/2023). Chickens were handled by trained personnel, housed under controlled biosafety conditions, and monitored daily to ensure animal welfare. All procedures complied with institutional policies and internationally recognized guidelines for the ethical use of animals in research.

### Study period and location

This study was conducted between July 2023 and February 2024 at the Biosafety Level 2 Animal Research Facility, Virology Laboratory, and Histopathology Laboratory, all located within the Faculty of Veterinary Medicine, Universiti Putra Malaysia, and at the Abadiah Laboratory, MTDC Technology Center III, Universiti Putra Malaysia.

### Experimental design and animal management

Sixty 21-day-old SPF chickens were housed in stainless steel wire cages within a Biosafety Level-2 Research Facility maintained at 26°C, 63% relative humidity, and continuous lighting, with feed and water provided ad libitum. The chickens were randomly allocated into Group A (nVarIBDV-inoculated, n = 28) and Group B (control, n = 32). Each group was maintained in separate rooms that were cleaned daily, with personnel movement from the control to the inoculated group and the use of disposable PPE and strict biosecurity measures.

At 0 dpi (21 days of age), chickens in Group A were inoculated with 1.0 mL of pathogenic field strain nVarIBDV (106.75 EID_50_/mL) via combined ocular (0.1 mL) and oral (0.9 mL) routes, while Group B remained uninoculated. Clinical signs were monitored at least twice daily, and any moribund chickens were humanely sacrificed for sampling. Four chickens from Group B were sacrificed at 0 dpi for baseline evaluation. Subsequently, four chickens from each group were randomly selected and sacrificed at 1, 3, 5, 7, 10, 14, and 21 dpi.

**Figure 1:**
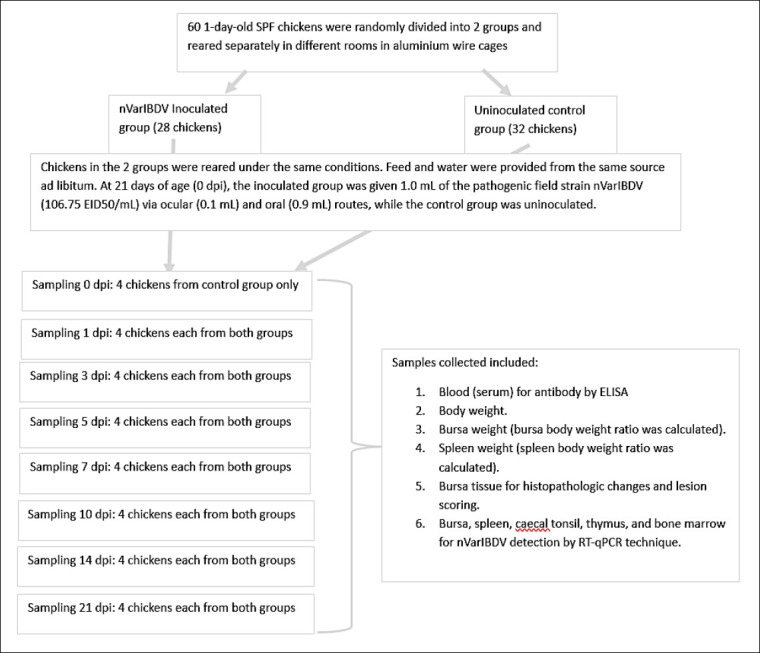
Schematic of the pathogenicity and immunogenicity timeline of SPF chickens inoculated with nVarIBDV. SPF = Specific-pathogen-free, nVarIBDV = Novel variant infectious bursal disease virus, dpi = Days post-inoculation, ELISA = Enzyme-linked immunosorbent assay, RT-qPCR = Reverse transcription quantitative polymerase chain reaction, EID_50_ = 50% Embryo infectious dose, mL = Milliliter, SPF = Specific-pathogen-free, nVarIBDV = Novel variant infectious bursal disease virus, dpi = Days post-inoculation, ELISA = Enzyme-linked immunosorbent assay, RT-qPCR = Reverse transcription quantitative polymerase chain reaction, EID50 = 50% Embryo infectious dose, mL = Milliliter.

Prior to sacrifice, body weight was recorded, and blood samples were collected for the determination of IBD antibody titers using ELISA. Gross lesions were recorded at necropsy. Bursa of Fabricius and spleen weights were measured, and bursa-to-body weight and spleen-to-body weight ratios were calculated. Bursa samples were fixed in Formalin solution (10% neutral buffered formalin) (SysterM, Shah Alam, Malaysia) for histopathology and lesion scoring. Additional samples from the bursa, spleen, cecal tonsil, thymus, and bone marrow were collected for the detection of nVarIBDV using RT-qPCR (Figure 1).

### nVarIBDV inoculum preparation

The nVarIBDV isolate (UPM1432/2019) was obtained from a commercial broiler farm in Selangor, Malaysia [3]. The isolate was confirmed as nVarIBDV by PCR and sequencing (GenBank accession number MT431217). Bursa tissues from affected chickens were processed, and the viral supernatant was inoculated into embryonated SPF chicken eggs. The CAM from infected eggs was harvested, mixed with PBS (Sigma-Aldrich, Missouri, USA) at a 1:2 ratio (CAM:PBS), manually homogenized with a mortar and pestle, and centrifuged at 252 × *g* for 5 min. The clarified supernatant was filtered through syringe filters (Membrane Solutions, Shanghai, China) to obtain the viral inoculum [19].

Viral titration was performed using the Reed and Muench method to determine EID_50_/mL [20]. The inoculum was stored at −80°C and used within 48 h after thawing.

### Virus titration

Ten-fold serial dilutions of nVarIBDV were inoculated into 9–11-day-old SPF embryonated chicken eggs, with five eggs per dilution, via the CAM route. Eggs were incubated at 37°C for 7 days and candled daily. Mortality within 24 h post-inoculation was regarded as nonspecific contamination and excluded from analysis; contaminated eggs were discarded [21]. Viral titers were calculated and expressed as EID_50_/mL using the Reed and Muench method [20].

### IBD antibody titer determination (ELISA)

Serum samples were analyzed for IBD antibody using a commercial IBDV antibody ELISA kit (BioChek BV, Reeuwijk, Netherlands). Antigen-coated plates were equilibrated to room temperature (22°C–27°C). Negative and positive controls (100 µL each) and 1:500 (v/v) diluted serum samples (100 µL) were added to the wells and incubated for 30 min at room temperature. Plates were washed four times with wash solution (350 µL/well), followed by the addition of 100 µL conjugate reagent (anti-chicken IgG conjugated with alkaline phosphatase) and incubation for 30 min.

After washing, 100 µL of substrate buffer was added to each well and incubated in the dark for 15 min at room temperature (22°C–27°C). The reaction was stopped by adding 100 µL stop solution. Absorbance was measured at 405 nm using a microplate reader, MR7000 (Dynatech Laboratories, Denkendorf, Germany), and IBD antibody titers were calculated using BioChek 2000 software.

### Viral load quantification by RT-qPCR

Pooled tissue samples from the bursa of Fabricius, spleen, thymus, bone marrow, and cecal tonsil were analyzed for viral detection using RT-qPCR in compliance with MIQE guidelines. RNA was extracted using a commercial RNA/DNA purification kit (Kylt®, Höltinghausen, Germany), with 20 mg of tissue per extraction. Lysis, binding, washing, and elution steps were performed according to the manufacturer’s instructions.

RT-qPCR was conducted using a SensiFAST™ Probe No-ROX One-Step Kit (Bioline, London, United Kingdom). Reverse transcription and amplification were performed using specific primers and probes (Table 1) [11]. Viral genome copy numbers were quantified using a Bio-Rad CFX Opus 96 real-time PCR system (BIO-RAD, Hercules, California, USA).

**Table 1:** Primers and probes for detecting and quantifying the novel variant infectious bursal disease virus in the tissues of inoculated specific-pathogen-free chickens.

Primers	Sequence (5′–3′)	Position (5′–3′)	Amplicon efficiency	Product size
F1432	CCAACAAGGGAGTACACCGA	1372–1391	99.8%	86 (base pairs)
R1432	CCAAATGCTCCTGCAATCTT	1438–1457		
Probe	AGTACTTCATGGAGGTGGCCGACCTCAA	1400–1427		

Designed from the sequence of the novel variant infectious bursal disease virus isolate UPM1432/2019 [11].

### Histopathology and bursal lesion scoring

Bursa of Fabricius samples were fixed in 10% buffered formalin (SysterM) for at least 24 h, trimmed to 5 mm thickness, and processed using an automated tissue processor (Leica ASP300, Wetzlar, Germany). Paraffin-embedded tissues (Leica EG1160) were sectioned at 4 µm and stained with hematoxylin and eosin (Leica). Histopathological changes were evaluated under light microscopy (Leica DM LB2).

Bursal lesion severity was scored in a blinded manner on a scale of 0–5 as follows: 0 = normal, 1 = mild, 2 = mild to moderate, 3 = moderate, 4 = moderate to severe, and 5 = severe [22].

### Statistical analysis

Data were analyzed using Student’s t-test in IBM SPSS Statistics version 23.0 (IBM Corp., Armonk, NY, USA). Results were expressed at a 95% confidence level, and differences were considered statistically significant when p < 0.05.

## RESULTS

### Clinical signs

No clinical signs were observed in Group B chickens throughout the study period. In contrast, chickens in Group A exhibited whitish to yellowish watery droppings from 3 to 12 dpi (Supplementary Figure 1), brownish to reddish watery droppings on 8, 9, and 18 dpi (Supplementary Figure 2), and ruffled feathers from 13 to 21 dpi (Supplementary Figure 3).

### Body weight changes

**Figure 2:**
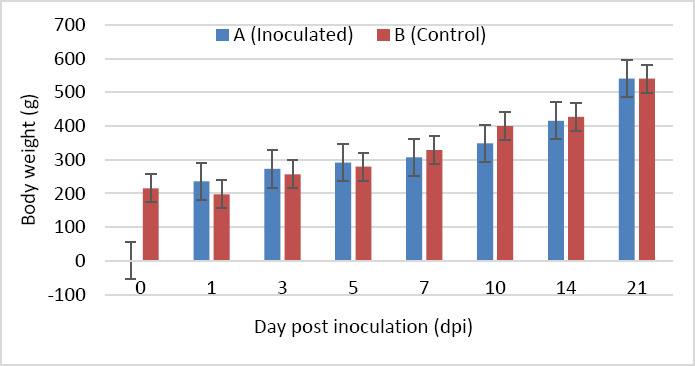
Body weight of SPF chickens inoculated with novel variant IBDV (n = 4 at each time point).

Body weight increased progressively in both groups from 0 to 21 dpi, with no statistically significant difference between groups at any time point (p > 0.05). However, chickens in Group A showed higher body weight at 1, 3, and 5 dpi, whereas chickens in Group B exhibited higher body weight at 7, 10, and 14 dpi (Figure 2).

### Bursa weight

The bursa weight of Group B increased steadily from 0 to 21 dpi. In contrast, Group A showed a marked reduction in bursa weight from 1 to 5 dpi, followed by stabilization from 5 to 21 dpi. No significant difference was observed between groups at 1 and 3 dpi (p > 0.05). However, from 5 to 21 dpi, bursa weight in Group A was significantly lower than in Group B (p < 0.05) (Supplementary Figure 4).

### Bursa-to-body weight ratio

**Figure 3:**
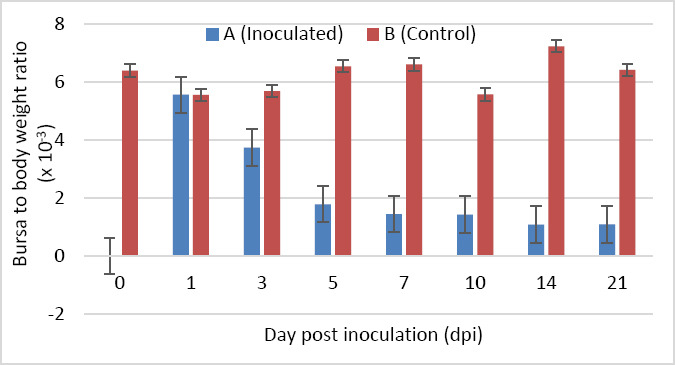
Bursa-to-body weight ratio of SPF chickens inoculated with novel variant IBDV (n = 4 at each time point).

No statistically significant difference was detected in the bursa-to-body weight ratio between groups at 1 dpi (p > 0.05). From 3 to 21 dpi, the ratio in Group A was significantly lower than in Group B (p < 0.05). In Group A, the bursa-to-body weight ratio declined sharply from 1 to 5 dpi and decreased more gradually from 5 to 21 dpi (Figure 3).

### Spleen weight

Spleen weight increased progressively in both groups from 1 to 21 dpi. At 5 and 7 dpi, spleen weight in Group A was significantly higher than in Group B (p < 0.05). At 21 dpi, spleen weight in Group A remained higher than in Group B, although the difference was not statistically significant (p > 0.05) (Supplementary Figure 5).

### Spleen-to-body weight ratio

**Figure 4:**
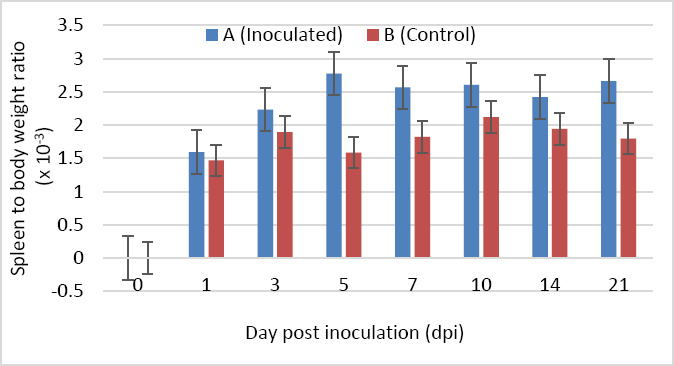
Spleen-to-body weight ratio of SPF chickens inoculated with novel variant IBDV (n = 4 at each time point).

The spleen-to-body weight ratio was higher in Group A than in Group B at 1, 3, 10, 14, and 21 dpi; however, these differences were not statistically significant (p > 0.05). In contrast, at 5 and 7 dpi, the ratio was significantly higher in Group A compared with Group B (p < 0.05) (Figure 4).

### Gross lesions

#### Bursa of Fabricius

**Figure 5:**
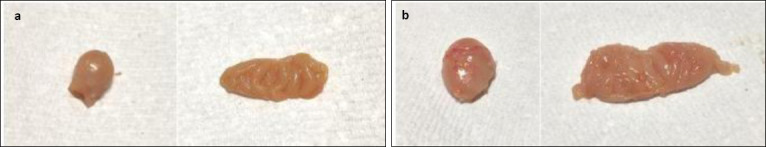
Gross bursal lesion of chickens on 10 dpi. (a) Group A, yellowish bursal atrophy bursa staining, decreased bursal folds, and firm consistency. (b) Group B, normal bursa.

**Figure 6:**
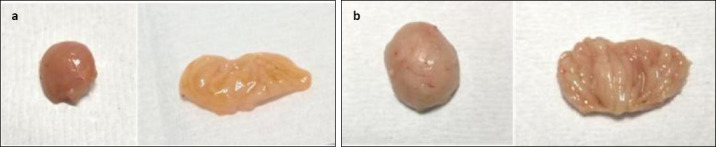
Gross bursal lesion of chickens on 21 dpi. (a) Group A, bursal atrophy, bursa yellowish staining, decreased bursal folds, and firm consistency. (b) Group B, normal bursa.

In Group A, gross bursal lesions were evident from 3 to 21 dpi and included bursal atrophy (3, 5, 7, 10, 14, and 21 dpi), yellowish discoloration (1, 3, 5, 7, 10, 14, and 21 dpi), reduced bursal folds (3, 5, 7, 10, 14, and 21 dpi), and firm bursal consistency (5, 7, 10, 14, and 21 dpi) (Figures 5a and 6a). No abnormal gross lesions were observed in the bursa of Group B chickens from 0 to 21 dpi (Figures 5b and 6b).

Spleen

**Figure 7:**
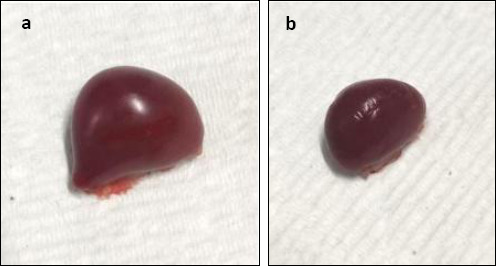
Gross lesion of the spleen of chickens on 7 dpi. (a) Splenomegaly in Group A. (b) Group B, normal spleen.

In Group A, splenomegaly was observed at 5 and 7 dpi compared with Group B (Figure 7a). No abnormal gross lesions were observed in the spleen of Group B throughout the trial period (Figure 7b).

Thymus

**Figure 8:**
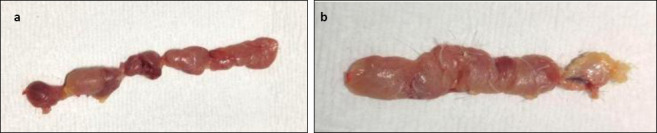
Gross lesion of the thymus of chickens on 21 dpi showing (a) reduced thymus size in Group A and (b) normal thymus size in Group B.

No gross lesions were observed in the thymus of either group from 1 to 7 dpi. From 10 to 21 dpi, the thymus of Group A appeared reduced in size compared with that of Group B (Figure 8).

### Histological lesions

#### Bursa of Fabricius

**Figure 9:**
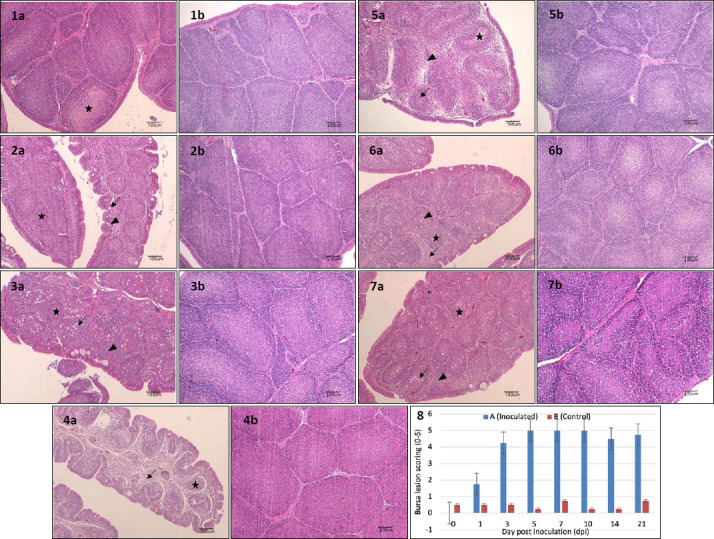
Histopathology of the bursa of Fabricius in SPF chickens inoculated with novel variant IBDV on 1 dpi. Images 1a–7a represent images of infected bursa from 1-21 dpi with lesion scoring of 4-5, 1b-7b represents the control with lesion scoring of 0-1, while image 8 shows the histogram of the bursa lesion scoring. Images 1a and 2a show moderate to severe lymphoid depletion (star), 2a–7a show bursal follicular atrophy (star) and edematous thickened interstitial connective tissue with inflammatory infiltration (arrow), images 3a show epithelial cyst formation (arrow), and images 4a–6a show vacuolation in the medullary region (arrow). 1b-7b shows intact bursal tissues. HE, 100x, Bar=100µm.

No histological abnormalities were observed in Group B throughout the experiment (Figure 9, Images 1b–7b). In Group A, moderate lymphoid depletion was observed at 1 dpi (Figure 9, Image 1a). At 3 dpi, lymphoid depletion, bursal follicular atrophy, and edematous thickening of interstitial connective tissue with inflammatory infiltration were evident (Figure 9, Image 2a). At 5 dpi, bursal follicular atrophy, epithelial cyst formation, and thickened, corrugated epithelium were observed (Figure 9, Image 3a). At 7 dpi, bursal follicular atrophy, thickened interstitial areas with inflammatory cell infiltration, and vacuolation in the medullary region were evident (Figure 9, Image 4a). Severe medullary vacuolation, interstitial thickening, inflammatory infiltration, and fibrous tissue proliferation were observed at 10 and 14 dpi (Figure 9, Images 5a and 6a). At 21 dpi, marked bursal follicular atrophy, interstitial thickening, inflammatory infiltration, and fibrous tissue proliferation persisted (Figure 9, Image 7a).

#### Bursal lesion scoring (0–5)

The bursal lesion score in Group B remained constant at 1 throughout the experiment. In contrast, the score in Group A increased sharply from 1 to 3 dpi, rose gradually from 3 to 5 dpi, remained stable until 10 dpi, declined slightly at 14 dpi, and increased again at 21 dpi. No significant difference was observed between groups at 1 dpi (p > 0.05). From 3 to 21 dpi, bursal lesion scores in Group A were significantly higher than in Group B (p < 0.05) (Figure 9, Image 8).

### IBD antibody response (ELISA)

**Figure 10:**
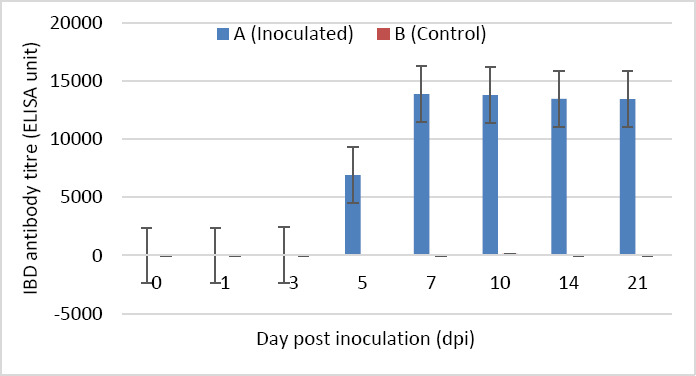
IBD antibody titer of SPF chickens inoculated with novel variant IBDV (n = 4 at each time point). Different letters (a and b) indicate significant differences between the groups (p < 0.05).

IBD antibodies became detectable from 5 dpi. Antibody titers increased rapidly between 5 and 7 dpi and gradually declined from 7 to 21 dpi. No statistically significant difference was observed between groups from 1 to 5 dpi (p > 0.05). From 7 to 21 dpi, antibody titers in Group A were significantly higher than those in Group B (p < 0.05) (Figure 10).

### Viral load distribution in organs

**Figure 11:**
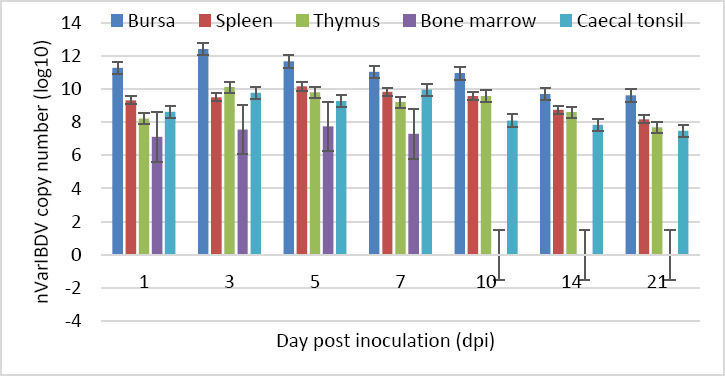
Viral loads in each organ of Group A chickens throughout the study.

The highest nVarIBDV load was detected in the bursa, whereas the lowest viral load was observed in the bone marrow. Viral loads in the bursa and thymus peaked at 3 dpi and gradually declined thereafter. Viral loads in the spleen and bone marrow peaked at 5 dpi, while the cecal tonsil reached the highest viral load at 7 dpi. All organs exhibited peak viral loads between 3 and 7 dpi. Viral RNA was not detected in the bone marrow after 7 dpi (Figure 11; Table 2).

**Table 2:** Viral loads of various organs in specific-pathogen-free chickens inoculated with the novel variant infectious bursal disease virus throughout the trial (n = 4 at each time point).

Organs	Log10 observed reaction (copies/titer)						

	Days post-inoculation (dpi)						

	1	3	5	7	10	14	21
BF	11.286	12.414	11.668	11.037	10.955	9.702	9.623
Spleen	9.327	9.506	10.170	9.836	9.573	8.748	8.178
Thymus	8.214	10.109	9.800	9.213	9.583	8.613	7.694
BM	7.111	7.557	7.745	7.298	ND	ND	ND
CT	8.627	9.767	9.266	9.971	8.102	7.839	7.484

BF = Bursa of Fabricius, BM = Bone marrow, CT = Cecal tonsil, ND = Not detected.

## DISCUSSION

### Overview of nVarIBDV pathogenicity

Since its initial description, nVarIBDV has spread widely across multiple regions and has been associated with immunosuppression and substantial economic losses in poultry production systems [17, 23]. In the present study, the pathogenicity and immunogenicity of nVarIBDV were comprehensively evaluated using 11 biological, pathological, immunological, and molecular parameters. The controlled experimental infection of SPF chickens using a defined inoculum dose (106.75 EID_50_/mL), dual inoculation routes, and systematic sampling intervals provides a robust and reproducible model for studying nVarIBDV infection dynamics.

### Clinical manifestations and production impact

Infected chickens exhibited ruffled feathers, watery diarrhea ranging from whitish–yellowish to brownish–red, depression, insensitivity, and reduced feed intake persisting up to 21 dpi, without recorded mortality. These findings indicate that nVarIBDV does not directly induce mortality but can cause prolonged clinical illness that may translate into significant economic losses due to reduced performance and increased management costs [4, 11, 23].

### Effects on growth performance

Although no statistically significant difference in body weight was observed between groups throughout the study, chickens in the control group had higher body weight at 7, 10, and 14 dpi. This suggests that nVarIBDV transiently impaired growth performance in Group A until 14 dpi, followed by recovery at 21 dpi. These findings differ from reports by Huang et al. [23] and Fan et al. [10], who observed significant body weight reduction following nVarIBDV infection. This discrepancy may be attributed to the use of SPF layer-type chickens in the present study, which inherently exhibit slower growth rates than commercial broilers.

### Bursa atrophy and immunosuppression

A significant reduction in bursa weight was observed in nVarIBDV-infected chickens from 5 to 21 dpi, indicating progressive bursal atrophy. These findings are consistent with previous reports showing that nVarIBDV induces marked bursal damage beginning at 5 dpi, with sustained effects [4–6, 11]. The bursa-to-body weight ratio further confirmed the occurrence and severity of bursal atrophy and served as a more reliable indicator of bursal damage. Bursal atrophy is a hallmark of IBDV-induced immunosuppression and predisposes infected chickens to secondary infections and vaccination failure. Indeed, nVarIBDV has been reported to reduce the efficacy of ND and AI vaccination [4, 10].

### Splenic response and immune competence

Splenomegaly was observed in nVarIBDV-infected chickens at 5 and 7 dpi, in agreement with previous findings [4]. However, other studies have reported no significant differences in spleen-to-body weight ratio between infected and control chickens [11, 23]. Histological alterations in the spleen may impair immune function by affecting lymphoid architecture and splenocyte activity, thereby contributing to overall immunosuppression [11].

### Gross and histopathological bursal lesions

Severe gross lesions, including bursal atrophy, yellowish discoloration, reduced folds, and firm consistency, were evident from 3 to 21 dpi, indicating extensive bursal damage following nVarIBDV infection. Histopatho-logically, moderate to severe lesions persisted throughout the same period, consistent with earlier reports [4, 11]. The observed lesions closely resemble those induced experimentally by vvIBDV [4, 11, 24]. Although vvIBDV is typically associated with high mortality, the comparable degree of bursal destruction observed with nVarIBDV highlights that immunosuppression alone can be equally detrimental to flock health and productivity.

### Humoral immune response dynamics

The humoral immune response was characterized by detectable IBD antibody production beginning at 5 dpi, with a rapid increase peaking at 7 dpi, coinciding with the active phase of viral replication. Immunosuppression following IBDV infection is primarily mediated by viral targeting of B lymphocytes and macrophages [25]. Additionally, effects on T cells [26] and splenocytes [27] have been reported, which may further compromise vaccine-induced immunity. Despite these immunosuppressive effects, high antibody titers (13,000–14,000) were maintained from 7 to 21 dpi, consistent with previous studies [11, 28, 29], indicating strong humoral immunogenicity and confirming the pathogenic potential of the isolate.

### Viral tissue tropism and persistence

Following oral entry, nVarIBDV initially replicates in GALT before entering the bloodstream and reaching the bursa, where extensive replication occurs, followed by secondary viremia and dissemination to other lymphoid organs [30]. Detection of viral RNA in the bursa, spleen, cecal tonsil, thymus, and bone marrow up to 21 dpi underscores the strong immunocompromising potential of nVarIBDV. To the best of our knowledge, this is the first report documenting nVarIBDV persistence in these organs for up to 21 dpi. Previous studies reported viral detection up to 14 dpi [31] or limited to the bursa up to 7 dpi [11], while other variant strains were described as short-lived and undetectable after 7 dpi [12, 32]. The sustained lesion severity observed from 3 dpi onward, coinciding with peak viral load in the bursa, indicates limited tissue recovery. Persistent infection of multiple immune organs may disrupt their structural integrity and function, facilitate viral shedding, and promote disease spread, thereby informing containment strategies and vaccination programs for ND and AI. Prolonged viral persistence may also exacerbate long-term immunosuppression and production losses, highlighting the importance of optimizing vaccination timing.

### Implications for vaccine development and future research

Given the ability of nVarIBDV to escape existing IBD vaccine immunity [3], the development of vaccines specifically targeting nVarIBDV is imperative. Potential approaches include whole-virus inactivated vaccines or recombinant vaccines expressing the VP2 protein using platforms such as HVT. Future studies should investigate the relationship between viral persistence and unique VP2 motifs, replication kinetics, and host immune responses. Molecular profiling of immune markers and cytokine expression, along with larger sample sizes, would further strengthen understanding of nVarIBDV pathogenesis. As viral RNA was detected up to 21 dpi, extended persistence studies may provide critical insights for refining containment measures and vaccine design.

## CONCLUSION

This study demonstrated that nVarIBDV is highly pathogenic and immunogenic in SPF chickens following controlled experimental infection. The virus induced persistent clinical signs without mortality, marked bursal atrophy from 5 to 21 dpi, transient splenomegaly, and severe gross and histopathological lesions in the bursa of Fabricius. High bursal lesion scores (4–5) were associated with peak viral loads between 3 and 7 dpi. Despite pronounced immunosuppression, strong humoral immune responses were observed, with high IBD antibody titers detected from 7 to 21 dpi. Importantly, nVarIBDV RNA was detected in multiple immune organs, including the bursa, spleen, cecal tonsil, thymus, and bone marrow, with persistence up to 21 dpi, indicating extensive tissue tropism and prolonged viral presence.

The prolonged persistence of nVarIBDV in primary and secondary lymphoid organs highlights its strong immunosuppressive potential and explains its association with vaccination failure and increased susceptibility to secondary infections. These findings have direct implications for IBD control programs, particularly in regions where nVarIBDV is endemic. The extended window of viral replication and organ persistence underscores the need to reconsider vaccination timing against IBDV, ND, and AI to minimize immune interference. Furthermore, the demonstrated multi-organ tropism suggests that infected chickens may serve as prolonged sources of viral shedding, necessitating stricter biosecurity and surveillance measures.

A major strength of this study lies in the use of SPF chickens, which eliminated confounding effects of maternal antibodies and prior pathogen exposure. The use of a well-characterized field isolate, standardized inoculum dose, dual inoculation routes, and systematic sampling across multiple dpi provided a comprehensive assessment of nVarIBDV pathogenicity, immunogenicity, and tissue distribution. Integration of clinical, pathological, immunological, and molecular endpoints allowed robust interpretation of disease dynamics.

This study was conducted under controlled experimental conditions using SPF chickens, which may not fully replicate field conditions in commercial poultry systems. Cellular immune responses and cytokine profiles were not assessed, limiting insights into the mechanistic basis of immunosuppression. In addition, viral persistence beyond 21 dpi was not evaluated, and sample size constraints may have limited detection of subtle inter-organ differences.

Future studies should investigate longer-term persistence of nVarIBDV beyond 21 dpi and assess its impact on cellular immunity, cytokine expression, and vaccine responsiveness. Comparative studies involving broiler and layer chickens under field-like conditions would improve external validity. Molecular characterization of viral determinants, particularly VP2-associated motifs, and their relationship with tissue persistence and immune evasion should be prioritized. Evaluation of nVarIBDV-specific vaccines, including inactivated and recombinant platforms, is also warranted to address current vaccine escape.

In conclusion, nVarIBDV causes severe and sustained immunopathology in SPF chickens, characterized by extensive bursal damage, prolonged multi-organ viral persistence, and strong but potentially misleading humoral responses. These findings provide critical evidence explaining nVarIBDV-associated immunosuppression, vaccine failure, and production losses. The study offers a solid experimental framework for vaccine-challenge models and contributes essential knowledge for refining IBD control strategies and developing next-generation vaccines targeting emerging variant strains.

## DATA AVAILABILITY

The supplementary data can be made available from the corresponding author upon request.

## AUTHORS’ CONTRIBUTIONS

HB: Conceptualization, data collection, and supervision. MM and NMS: Data collection and analysis, supervision. BTH and CHH: Data collection and analysis and experimentation. UCC: Data collection and analysis, experimentation, supervision, writing- original draft, and writing- review and editing. All authors have read and approved the final version of the manuscript.

## COMPETING INTERESTS

The authors declare that they have no competing interests.

## PUBLISHER’S NOTE

Veterinary World remains neutral with regard to jurisdictional claims in the published institutional affiliations.
